# Selective decontamination in European intensive care patients

**DOI:** 10.1007/s00134-012-2488-1

**Published:** 2012-02-01

**Authors:** Evelien A. N. Oostdijk, Bastiaan H. J. Wittekamp, Christian Brun-Buisson, Marc J. M. Bonten

**Affiliations:** 1 Department of Medical Microbiology, University Medical Center Utrecht, G04.614, PO box 85500, 3508 GA Utrecht, The Netherlands; 2 Department of Intensive Care Medicine, University Medical Center Utrecht, Utrecht, The Netherlands; 3 Julius Center for Health Sciences and Primary Care, University Medical Center Utrecht, Utrecht, The Netherlands; 4 INSERM U955, Université Paris Est-Créteil, Créteil, France; 5 Service de Réanimation Médicale, Assistance Publique-Hôpitaux de Paris, Groupe Henri Mondor, Créteil, France; 6 Faculté de Médecine, Université Paris-Est, Creteil, France

## Introduction

Selective decontamination of the digestive tract (SDD) is both one of the most studied and one of the most debated preventive measures for critically ill patients in intensive care units (ICUs) (see box). After the first trials in hematology patients in the 1970s [1, 2], the concept was introduced in ICU populations in the 1980s [3], and frequently studied in the following decade [4]. Various different regimens were studied, including strictly oropharyngeal decontamination [selective oropharyngeal decontamination (SOD)] (see box). At the turn of the century there were more than 50 randomized, though mostly small and single-center, trials and several meta-analyses. The summarized conclusions from these studies were that SDD was associated with reductions of respiratory tract infections in ICUs with low levels of antibiotic resistance, but that improvement of patient outcome (i.e., better ICU survival) could be demonstrated in meta-analyses only [5, 6].

Since that time, numbers of new SDD studies declined and this measure was not widely adopted in European ICUs, mainly because the evidence for better patient outcome was considered not convincing, and because of the unknown—possibly detrimental—effects of prophylactic antibiotic use on antibiotic resistance development. The Netherlands became the exception to this rule, due to two studies, both demonstrating survival benefits of patients receiving SDD [7, 8]. In both studies, SDD was used as a unit-wide intervention in ICUs with low prevalence of antibiotic-resistant bacteria, and in both studies SDD was associated with lower, instead of higher, rates of antibiotic resistance. However, the absolute 28-day mortality reduction in the largest study was 3.5% (relative reduction was 13%) and only determinable in a random-effects logistic regression model with adjustment for baseline differences between study groups [8]. Moreover, in the latter study, SDD was equally effective in improving patient outcome as SOD.

The beneficial results of SDD and SOD obtained in Dutch ICUs raise the question of whether both measures could be equally beneficial in other European countries. Here, we address some methodological issues relevant to future attempts to quantify the effects of SDD or SOD in critically ill patients.

## Study design

The two studies in which SDD was associated with improved patient outcome tested SDD as a unit-wide intervention. In one study, SDD was administered to all patients who provided informed consent in one ICU during a 2-year study period (*n* = 466), and results were compared with those of another ICU (in the same hospital) where none of the patients received SDD (*n* = 468) [7]. Allocation of patients to either of the two wards was randomized if both units had beds available, but this was not further specified. In the other study, SDD was also administered to all patients eligible during a 6-month period in 13 ICUs (*n* = 2,045), and the results were compared with those obtained in 6-month periods in which all patients received either SOD (*n* = 1,904) or no topical antibiotics (*n* = 1,990) [8]. The latter was—at that time—considered standard of care. In this cluster-randomized crossover study, each of the 13 ICUs used SDD, SOD, and standard care during 6-month periods, with the order of interventions randomized per center. Importantly, in the latter study, there was no individualized randomization, which bears the risk, intrinsic to any cluster-randomized study, of biased patient inclusion [9]. Therefore, baseline characteristics related to patient prognosis must be included in the analysis.

There is an obvious reason to evaluate SDD and SOD as a unit-wide measure. Both measures aim to reduce bacterial carriage in individual patients, which may influence the risk of acquisition of bacterial colonization (followed by infection) of other patients. This patient dependency might reduce the true effects of interventions when patients with and without SDD (or SOD) are treated in the same unit [10]. As a result, failure to demonstrate beneficial outcome results in an individual patient randomized study may not reflect true effects when using these measures in all patients.

## Outcome measures

A number of outcomes can be measured when studying infection prevention strategies in the ICU, including infection rates and antibiotic use, length of stay or of mechanical ventilation, and mortality rates in the ICU or at a fixed time-point (e.g., 28 days), or ventilator-free days (surviving) at 28 days. Which of these is most appropriate as the primary end-point in decontamination studies? It is widely believed that SDD and SOD exert their effects largely through prevention of respiratory tract infections, such as ventilator-associated pneumonia (VAP). As compared with SOD, the intestinal decontamination part of SDD seems to reduce the occurrence of ICU-acquired bacteremia with Gram-negative bacilli, but it is unlikely that this effect can be determinable in survival differences [11]. Diagnosing VAP is difficult and relies for an important part on microbiological culture results from respiratory samples [12]. The topical antibiotics applied in the oropharynx, though, aim to eradicate bacterial colonization of the upper respiratory tract, which will inevitably influence culture results. Only samples obtained from the distal parts of the lung that cannot be reached by the topical antibiotics will provide reliable diagnostic samples. Therefore, unambiguous, patient-centered outcomes such as survival should be used as end-points when evaluating these interventions. Moreover, since recent studies have convincingly demonstrated that the attributable mortality of VAP is much lower than previously assumed, it is difficult to extrapolate a reduction in VAP incidence to improved patient survival [13–15].

What should be the targeted mortality reduction? The relative reduction in 28-day mortality in the Dutch multicenter study was 13% for SDD and 11% for SOD, corresponding to absolute mortality reductions at day 28 of 3.5% and 2.7%, respectively [8]. Based on these estimates, derived in units with low levels of antibiotic resistance, at least 2,000 patients per intervention group are needed to gain sufficient power. However, it would be highly relevant to determine outcome effects of these interventions on longer time scales, such as 90-day or 1-year survival, which may well enhance the number of patients needed. Furthermore, it is difficult to anticipate the magnitude of the effect on patient outcome in settings with different bacterial ecology, i.e., with higher prevalence of antibiotic-resistant bacteria. If the preventive effects on infection development are similar in such a setting, but attributable mortality of infection is higher because of more infections being caused by antibiotic-resistant bacteria, the effects on patient survival could be larger than those obtained in Dutch ICUs. In contrast, if fewer infections are prevented because of antibiotic resistance, it can be expected that effects on survival will be smaller (or even absent).

## Antibiotic resistance

The global emergence of antibiotic resistance, especially among Enterobacteriaceae, necessitates enhanced infection control strategies, also in ICUs. In theory, SDD and SOD could have a synergistic effect with basic infection control measures such as hand hygiene and barrier precautions. Reductions of bacterial loads at places frequently contacted by nursing staff (i.e., the respiratory tract region), would reduce the likelihood of cross-transmission. However, the evidence on the effects of SDD on antibiotic resistance is highly conflicting. In settings with low levels of antibiotic resistance, such as Dutch ICUs, SDD and SOD were associated with lower rates of antibiotic-resistant Gram-negative bacteria [7, 16], and ongoing follow-up studies seem to confirm these findings (M. Bonten, unpublished data).

Less certain are the effects of topical antibiotics in settings with higher levels of resistance. There is—already “old”—evidence that SDD can help to control outbreaks with multiresistant *Klebsiella* strains [17], and persistently low levels of resistance have been reported from several centers using SDD for prolonged periods of time. In contrast, in some studies, use of SDD was associated with increasing rates of carriage and infections caused by antibiotic-resistant (mostly Gram-positive) pathogens [18–21].

Colistin is one of the antibiotics used in SDD and SOD. The recent rise of infections caused by carbapenem-resistant Gram-negative bacteria makes this agent a last-resort antibiotic. It is therefore imperative to determine the effects of topical use of colistin on resistance development in Gram-negative bacteria. Furthermore, it is unknown whether patients recolonize with resistant bacteria when SDD is discontinued. Another aspect related to antibiotic resistance is the total amount of intravenous antibiotic use. According to the “classical” SDD protocol, all patients should receive intravenous antibiotics during the first 4 days. However, systemic antibiotics are also prescribed to virtually all eligible ICU patients, whether or not they are receiving SOD or no topical prophylaxis at all [8]. In the Dutch multicenter study, the total use of intravenous antibiotics (including the SDD component) was around 10% lower during SDD and SOD [8]. It is unknown to what extent such a reduction in systemic antibiotic use may influence resistance development.

## Other considerations

A formal cost–benefit analysis of SDD does not exist. In the Dutch multicenter study it was estimated that the daily antibiotic costs of SDD and SOD were US $12 and US $1, respectively [8]. However, especially the price of amphotericin B has markedly risen in recent years. Today, the commercial price of SDD and SOD would be around €200 and €40 per day, respectively. Since the necessity of amphotericin B as a component of SDD has never been determined, it might be worthwhile to investigate SDD with other topical antimycotic agents (e.g., nystatin) [22–25].

Based on the Dutch multicenter study, one could conclude that SOD and SDD are equally effective in improving patient outcome. If confirmed, this implies that the improved outcome essentially results from the effects of the strategy on oropharyngeal bacterial carriage. Chlorhexidine oropharyngeal care has also been associated with a 50% reduction in VAP [26], quite similar to the reported effects of SOD [27], but the two interventions have never been compared directly. A recent meta-analysis suggests a dose–response relationship with optimal preventive effects of chlorhexidine oropharyngeal care when using a concentration of 2% [28]. If chlorhexidine is indeed equally effective as SDD and SOD, it would overcome all the potential problems with using topical antibiotics for prophylaxis in critically ill patients.

## Future studies

Currently, there is only one large randomized clinical trial registered which evaluates the effects of SDD or SOD, again in Dutch ICUs (Table 1). Based on the favorable results obtained in Dutch ICUs, the logical next step seems to investigate SDD and SOD in settings with different bacterial ecology. However, when preparing such studies, some important lessons can be learned from the former studies. The beneficial effects of the interventions tested in individual studies have only been apparent when applied as unit-wide interventions. It is, therefore, advisable to apply this approach in any further study. Because of the difficulties in objectively diagnosing VAP and the fact that SDD and SOD cannot be applied in a double-blind manner, it is advisable to use an unambiguous primary outcome, such as patient survival. Considering the small absolute reduction in 28-day mortality derived in Dutch ICUs, study groups should include at least 2,000 subjects. Detailed monitoring of antibiotic resistance is imperative, especially for colistin resistance; finally, studies comparing these interventions with 2% chlorhexidine oropharyngeal care are warranted.

**Table 1:** Registered trials (http://www.clinicaltrials.gov, M. Bonten, personal communication)

Study	Year	Institute	Patients	Design	Primary objective	Intervention	Study status
Microbiologic effect of selective decontamination of the digestive tract with colistin, gentamicin, and nystatin	2007	University of Pittsburgh Pennsylvania	40	Observational	Effect of SDD on vancomycin-resistant enterococci colonization	SDD (colistin, gentamicin, nystatin)	Not started, terminated
Selective digestive decontamination in carriers of carbapenem-resistant *Klebsiella pneumoniae*	2008	Soroka University Medical Center, Israel	*Unknown*	Blinded RCT	Effect of SDD on carbapenem-resistant *Klebsiella*	SDD (gentamicin and polymyxin E)	Completed
The effects of SDD and SOD on antibiotic resistance in the ICU	2009	University Medical Center Utrecht, Utrecht, The Netherlands	10,000	Randomized crossover multicenter trial	Effect of SDD and SDD on antimicrobial resistance	SDD and SOD (amphotericin B, tobramycin, colistin, and cefotaxim in SDD)	Running
Resistant Gram-negative bacteria after cessation of SDD or SOD in ICU patients	2010	Leiden University Medical Center, Leiden, The Netherlands	1,200	Prospective observational multicenter trial	Rectal colonization with any resistant aerobic Gram-negative bacteria at any time point within 10 days after ICU discharge	SDD and SOD (amphotericin B, tobramycin, colistin, and cefotaxim in SDD)	Running
R-GNOSIS: Decolonization strategies in intensive care	2011	University Medical Center Utrecht, Utrecht, The Netherlands	10,400	Randomized crossover multicenter trial	Effect of SDD, SOD, and chlorhexidine mouthwash on Gram-negative bacteremia rate	SDD and SOD (amphotericin B, tobramycin, colistin) and chlorhexidine 2%	Prior to participant recruitment



## References

[1] Dekker AW, Rozenberg-Arska M, Sixma JJ, Verhoef J (1981). Prevention of infection by trimethoprim-sulfamethoxazole plus amphotericin B in patients with acute nonlymphocytic leukaemia. Ann Intern Med.

[2] Rozenberg-Arska M, Dekker AW, Verhoef J (1983). Colistin and trimethoprim-sulfamethoxazole for the prevention of infection in patients with acute non-lymphocytic leukaemia. Decrease in the emergence of resistant bacteria. Infection.

[3] Stoutenbeek CP, van Saene HK, Miranda DR, Zandstra DF (1984). The effect of selective decontamination of the digestive tract on colonisation and infection rate in multiple trauma patients. Intensive Care Med.

[4] Bonten MJ, Kullberg BJ, van Dalen R, Girbes AR, Hoepelman IM, Hustinx W, van der Meer JW, Speelman P, Stobberingh EE, Verbrugh HA, Verhoef J, Zwaveling JH (2000). Selective digestive decontamination in patients in intensive care. The Dutch working group on antibiotic policy. J Antimicrob Chemother.

[5] D’Amico R, Pifferi S, Leonetti C, Torri V, Tinazzi A, Liberati A (1998). Effectiveness of antibiotic prophylaxis in critically ill adult patients: systematic review of randomised controlled trials. BMJ.

[6] Liberati A, D’Amico R, Pifferi S, Torri V, Brazzi L, Parmelli E (2009) Antibiotic prophylaxis to reduce respiratory tract infections and mortality in adults receiving intensive care. Cochrane Database Syst Rev CD000022 10.1002/14651858.CD000022.pub2 14973945

[7] de Jonge E, Schultz MJ, Spanjaard L, Bossuyt PM, Vroom MB, Dankert J, Kesecioglu J (2003). Effects of selective decontamination of digestive tract on mortality and acquisition of resistant bacteria in intensive care: a randomised controlled trial. Lancet.

[8] de Smet AM, Kluytmans JA, Cooper BS, Mascini EM, Benus RF, van der Werf TS, van der Hoeven JG, Pickkers P, Bogaers-Hofman D, van der Meer NJ, Bernards AT, Kuijper EJ, Joore JC, Leverstein-van Hall MA, Bindels AJ, Jansz AR, Wesselink RM, de Jongh BM, Dennesen PJ, van Asselt GJ, te Velde LF, Frenay IH, Kaasjager K, Bosch FH, van Iterson M, Thijsen SF, Kluge GH, Pauw W, de Vries JW, Kaan JA, Arends JP, Aarts LP, Sturm PD, Harinck HI, Voss A, Uijtendaal EV, Blok HE, Thieme Groen ES, Pouw ME, Kalkman CJ, Bonten MJ (2009). Decontamination of the digestive tract and oropharynx in ICU patients. N Engl J Med.

[9] Puffer S, Torgerson D, Watson J (2003). Evidence for risk of bias in cluster randomised trials: review of recent trials published in three general medical journals. BMJ.

[10] Nijssen S, Bootsma M, Bonten M (2006). Potential confounding in evaluating infection-control interventions in hospital settings: changing antibiotic prescription. Clin Infect Dis.

[11] Oostdijk EA, de Smet AM, Kesecioglu J, Bonten MJ (2011). The role of intestinal colonization with gram-negative bacteria as a source for intensive care unit-acquired bacteremia. Crit Care Med.

[12] Brun-Buisson C, Fartoukh M, Lechapt E, Honore S, Zahar JR, Cerf C, Maitre B (2005). Contribution of blinded, protected quantitative specimens to the diagnostic and therapeutic management of ventilator-associated pneumonia. Chest.

[13] Bekaert M, Timsit JF, Vansteelandt S, Depuydt P, Vesin A, Garrouste-Orgeas M, Decruyenaere J, Clec’h C, Azoulay E, Benoit D (2011) Attributable Mortality of Ventilator Associated Pneumonia: A Reappraisal Using Causal Analysis. Am J Respir Crit Care Med 184:1133–1139 10.1164/rccm.201105-0867OC 21852541

[14] Melsen WG, Rovers MM, Koeman M, Bonten MJ (2011). Estimating the attributable mortality of ventilator-associated pneumonia from randomized prevention studies. Crit Care Med.

[15] Nguile-Makao M, Zahar JR, Francais A, Tabah A, Garrouste-Orgeas M, Allaouchiche B, Goldgran-Toledano D, Azoulay E, Adrie C, Jamali S, Clec’h C, Souweine B, Timsit JF (2010). Attributable mortality of ventilator-associated pneumonia: respective impact of main characteristics at ICU admission and VAP onset using conditional logistic regression and multi-state models. Intensive Care Med.

[16] de Smet AM, Kluytmans JA, Blok HE, Mascini EM, Benus RF, Bernards AT, Kuijper EJ, Leverstein-van Hall MA, Jansz AR, de Jongh BM, van Asselt GJ, Frenay IH, Thijsen SF, Conijn SN, Kaan JA, Arends JP, Sturm PD, Bootsma MC, Bonten MJ (2011). Selective digestive tract decontamination and selective oropharyngeal decontamination and antibiotic resistance in patients in intensive-care units: an open-label, clustered group-randomised, crossover study. Lancet Infect Dis.

[17] Brun-Buisson C, Legrand P, Rauss A, Richard C, Montravers F, Besbes M, Meakins JL, Soussy CJ, Lemaire F (1989). Intestinal decontamination for control of nosocomial multiresistant gram-negative bacilli. Study of an outbreak in an intensive care unit. Ann Intern Med.

[18] de La Cal MA, Cerda E, Garcia-Hierro P, van Saene HK, Gomez-Santos D, Negro E, Lorente JA (2005). Survival benefit in critically ill burned patients receiving selective decontamination of the digestive tract: a randomized, placebo-controlled, double-blind trial. Ann Surg.

[19] Heininger A, Meyer E, Schwab F, Marschal M, Unertl K, Krueger WA (2006). Effects of long-term routine use of selective digestive decontamination on antimicrobial resistance. Intensive Care Med.

[20] Lingnau W, Berger J, Javorsky F, Fille M, Allerberger F, Benzer H (1998). Changing bacterial ecology during a five-year period of selective intestinal decontamination. J Hosp Infect.

[21] Verwaest C, Verhaegen J, Ferdinande P, Schetz M, Van den Berghe G, Verbist L, Lauwers P (1997). Randomized, controlled trial of selective digestive decontamination in 600 mechanically ventilated patients in a multidisciplinary intensive care unit. Crit Care Med.

[22] Cockerill FR, Muller SR, Anhalt JP, Marsh HM, Farnell MB, Mucha P, Gillespie DJ, Ilstrup DM, Larson-Keller JJ, Thompson RL (1992). Prevention of infection in critically ill patients by selective decontamination of the digestive tract. Ann Intern Med.

[23] Flaherty J, Nathan C, Kabins SA, Weinstein RA (1990). Pilot trial of selective decontamination for prevention of bacterial infection in an intensive care unit. J Infect Dis.

[24] Ruza F, Alvarado F, Herruzo R, Delgado MA, Garcia S, Dorao P, Goded F (1998). Prevention of nosocomial infection in a pediatric intensive care unit (PICU) through the use of selective digestive decontamination. Eur J Epidemiol.

[25] Wiener J, Itokazu G, Nathan C, Kabins SA, Weinstein RA (1995). A randomized, double-blind, placebo-controlled trial of selective digestive decontamination in a medical-surgical intensive care unit. Clin Infect Dis.

[26] Koeman M, van der Ven AJ, Hak E, Joore HC, Kaasjager K, de Smet AG, Ramsay G, Dormans TP, Aarts LP, de Bel EE, Hustinx WN, van der Tweel I, Hoepelman AM, Bonten MJ (2006). Oral decontamination with chlorhexidine reduces the incidence of ventilator-associated pneumonia. Am J Respir Crit Care Med.

[27] Bergmans DC, Bonten MJ, Gaillard CA, Paling JC, van der Geest S, van Tiel FH, Beysens AJ, de Leeuw PW, Stobberingh EE (2001). Prevention of ventilator-associated pneumonia by oral decontamination: a prospective, randomized, double-blind, placebo-controlled study. Am J Respir Crit Care Med.

[28] Labeau SO, Van de Vyver K, Brusselaers N, Vogelaers D, Blot SI (2011). Prevention of ventilator-associated pneumonia with oral antiseptics: a systematic review and meta-analysis. Lancet Infect Dis.

